# Rapid Advances in Understanding Viral Gastroenteritis from Domestic Surveillance 

**DOI:** 10.3201/eid1908.130449

**Published:** 2013-08

**Authors:** Daniel C. Payne, Umesh D. Parashar

**Affiliations:** Centers for Disease Control and Prevention, Atlanta, Georgia, USA

**Keywords:** viral gastroenteritis, enteric diseases, surveillance, viruses, acute gastroenteritis

“Winter vomiting disease” was the clinical moniker for viral acute gastroenteritis (AGE), including illnesses caused by norovirus and rotavirus, nearly 100 years ago (*1*). This nonspecific diagnosis represented a frequently observed illness, with the symptoms of vomiting and diarrhea that occurred particularly in the colder months. One hundred years later, diagnosing specific AGE pathogens in clinical settings continues to be an elusive task. Clinical treatment options are nonspecific as well—primarily rehydration and supportive therapies—and the identification of the viral pathogens is considered relatively time-consuming and costly.

In truth, viral AGE has likely caused misery, illness, and death among human populations for thousands of years, since people first facilitated disease transmission by congregating in groups. But, it has been only during the lifetime of many current readers (and during the long career spans of several), beginning in the early 1970s, that norovirus, rotavirus, and an expanding collection of other viral AGE pathogens have been discovered. The advent of sensitive laboratory tools to detect and study the genetic evolution of these viruses has uncovered their critical role in the etiology of AGE. The flow of information is now so great that in each year since 2008, >800 scientific papers have been published on this topic as determined by a search of PubMed using the term acute gastroenteritis.

The field of viral gastroenteritis is in the midst of an extraordinary period of rapid development and transition. Vaccines to prevent rotavirus, the leading cause of severe childhood AGE worldwide, are being rolled out globally and have already achieved remarkable success in reducing the burden of this pathogen in many countries, including the United States. In addition, the application of sensitive molecular assays is reaffirming the central etiologic role of noroviruses in both endemic and epidemic AGE, and vaccines against this pathogen are undergoing clinical testing. This issue of Emerging Infectious Diseases highlights recent developments in the field with a collection of timely findings from domestic viral gastroenteritis surveillance, which will further our understanding of disease effects, viral evolution and structure, implications of vaccination, and progress with other preventive measures.

**Figure:**
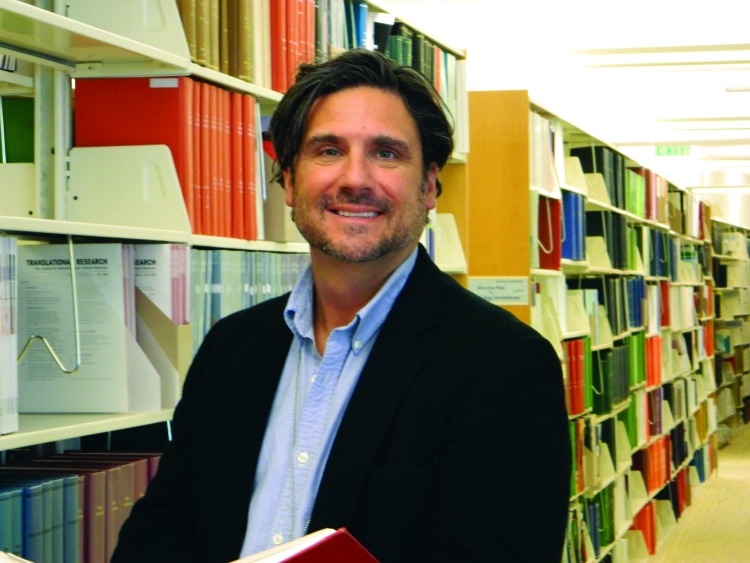
Daniel Payne.
